# Anti-cancer effects of nitrogen-containing bisphosphonates on human cancer cells

**DOI:** 10.18632/oncotarget.10773

**Published:** 2016-07-22

**Authors:** Pengfei Jiang, Peiying Zhang, Rajesh Mukthavaram, Natsuko Nomura, Sandeep C. Pingle, Dayu Teng, Shu Chien, Fang Guo, Santosh Kesari

**Affiliations:** ^1^ Moores Cancer Center, UC San Diego, La Jolla, CA 92093, USA; ^2^ Department of Cardiology, Xuzhou Central Hospital, The Affiliated XuZhou Hospital of Medical College of Southeast University, Xuzhou Clinical School of Xuzhou Medical College, Xuzhou Clinical Medical College of Nanjing University of Chinese Medicine, Xuzhou, Jiangsu Province 221009, China; ^3^ Department of Bioengineering, UC San Diego, La Jolla, CA 92093, USA; ^4^ Key Laboratory of Systems Biology, Shanghai Advanced Research Institute, Chinese Academy of Sciences, Shanghai 201210, China; ^5^ Department of Translational Neuro-Oncology and Neurotherapeutics, John Wayne Cancer Institute and Pacific Neuroscience Institute at Providence Saint John's Health Center, Santa Monica, CA 90404, USA; ^6^ Institute of Engineering in Medicine, UC San Diego, La Jolla, CA 92093, USA

**Keywords:** glioblastoma, bisphosphonates, zoledronic acid, cancer therapy, autophagy

## Abstract

Zoledronic acid, a potent nitrogen-containing bisphosphonate (NBP), has been extensively used to limit bone turnover in a various diseases including tumors. Recent clinical studies have demonstrated direct anti-cancer effects of zoledronic acid, in addition to its clinical benefits for skeletal-related events. Here we investigated the effects of 4 clinically available NBPs on human tumor cell proliferation. Our data demonstrate a potent anti-proliferative effect of zoledronic acid against glioblastoma (GBM) cell lines, breast cancer cells and GBM patient-derived lines. Zoledronic acid also effectively inhibited GBM tumor growth in xenograft mouse models. Zoledronic acid strongly stimulated autophagy but not apoptotic signals in all tested cells. Only one intermediate product of cholesterols synthesis pathway, geranylgeranyl diphosphate (GGPP) rescued cells from the cytotoxic effects of zoledronic acid. To further investigate the effect of GGPP, we knocked down RABGGTA, which encodes a subunit of the Rabgeranylgeranyltransferase protein. This knockdown induced an effect similar to zoledronic acid in cancer cell lines. These data are promising and suggested a potential for zoledronic acid as an anti-cancer agent, through its ablation of the function of Rab proteins.

## INTRODUCTION

Bisphosphonates (BPs) are synthetic analogues of pyrophosphate, that bind bone with high affinity. BPs exert cytotoxic effects on osteoclasts and slow down bone-resorption. BPs are the most commonly prescribed drugs used for the prevention and treatment of osteoporosis, osteitis deformans, primary hyperparathyroidism, osteogenesis imperfecta, and other conditions that feature bone fragility. BPs also has been used to reduce skeletal complications and pain in multiple myeloma and the bone metastatic phase of a variety of solid tumors, including breast [1], prostate [2, 3] and lung cancer [4]. However, recent evidence suggests that an added beneficial clinical effect in cancer patients may be related to direct antitumor activity of BPs [5, 6].

Nitrogen-containing bisphosphonates (NBPs) are second- and third-generation BPs, have greater anti-bone-resorptive potency (10-10,000 fold) than non-nitrogen-containing bisphosphonates (non-NBPs). NBPs include 7 FDA approved drugs: Pamidronate (APD, Aredia), Neridronate (Nerixia), Olpadronate, Alendronate (Fosamax), Ibandronate (Boniva), Risedronate (Actonel), and Zoledronate (Zometa). NBPs are more prescribed in clinical usage. Zoledronate (Zoledronic acid) is a NBP with highest activity against osteoclasts, when compared to other drugs [7]. Zoledronic acid can reduce and delay bone complications from bone metastases, and has been used in over 4 million patients worldwide for bone metastases from solid tumors and bone complications from multiple myeloma [8]. In the clinic, zoledronic acid as well as other NBPs have been demonstrated to have additional anticancer effects [9]. Clinical studies demonstrate that zoledronic acid improves disease-free survival, decreases residual invasive tumor size, and reduces circulating tumor cells. The possible mechanisms of zoledronic acid action include inhibition of angiogenesis factors such as VEGF, and inducing microenvironment changes that attenuate tumor growth [10, 11, 12].

It is important to investigate the direct anticancer effects of NBPs, independent of its effects on osteoclastic bone resorption. In the last decade, NBPs have been identified as potent inhibitors of critical enzymes of the mevalonate pathway. For example, zoledronic acid and risedronate can target farnesyl pyrophosphate synthase (FPPS) [13]; zoledronic acid is one of the most potent inhibitors of FPPS [14]; additionally, NBPs can bind and inhibit geranylgeranyl diphosphate synthase (GGPPS) and geranylgeranyl transferase I [15, 16, 17, 18]. Based on these effects, we hypothesized that NBPs are potent inhibitors of human cancer cell growth, since the enzymes and the mevalonate synthesis pathway targeted by NBPs are conserved in all mammalian cells including tumor cells.

Glioblastoma (GBM), grade IV astrocytoma, is the most common and aggressive malignant brain tumor in adults. Standard therapy for GBM consists of surgical resection, radiotherapy, and chemotherapy with temozolomide. Even following aggressive treatment, the tumor typically recurs within 1 year and patient survival rate is still very low [19, 20]. It is important to develop new approaches to treat this condition and improve overall survival, along with quality of life for patients and survivors. A new strategy termed CUSP9 has been proposed to repurpose nine already-marketed drugs, as adjuvants to improve effectiveness and tolerability of low-dose continuous temozolomide in treatment of recurrent glioblastoma [21]. Evaluating additional FDA approved drugs such as statin and NBPs for repurposing, will further improve this process and greatly facilitate fulfilling the unmet medical need for GBM patients. In this study, we aimed to determine if zoledronic acid can inhibit human cancer cell proliferation, in primary tumor cells derived from GBM patients as well as highly metastasizing cancer (breast cancer); we also elucidated the underlying molecular mechanisms of tumor cells death induced by NBPs. Interestingly, our rescue experiments suggest that the depletion of GGPP mediates the cytotoxic effects of zoledronic acid. More importantly, we provided a clear evidence that inhibition of RABGGTase but not GGTase-1 was the major mechanism for cancer cell death induced by NBPs. The results from our study provide feasibility for repurposing of NBPs for cancer therapy, and support further studies on druggable targets in the cholesterol synthesis pathway.

## RESULTS

### Zoledronic acid inhibits proliferation of breast cancer cells, GBM cells, and GBM spheres

**Figure 1:**
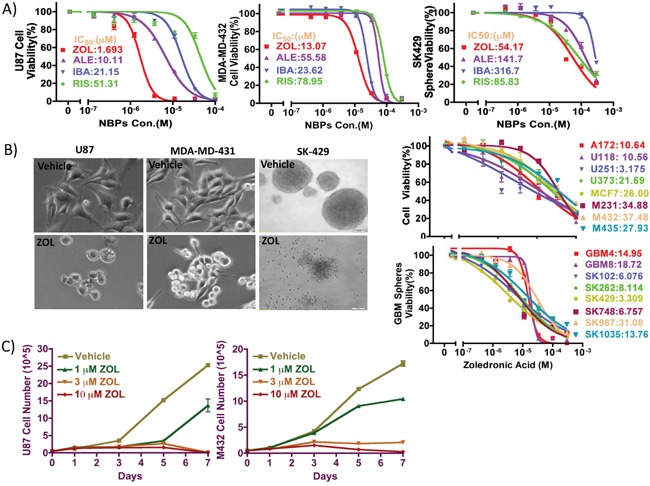
Inhibitory effect of NBPs against tumor cells proliferation *in vitro* **A.** The anti-proliferative effect of 4 clinically available NBPs was tested against against glioma cells, breast cancer cells, and patient-derived glioma neurospheres using alamar blue assay. ZOL showed the lowest IC_50_ values, as compared to other NBPs in all cell types tested. **B.** ZOL broadly inhibits cancer cell lines proliferation, as demonstrated by its effect on morphology and representative dose-response curves. **C.** To determine effect of ZOL on viability, cells were treated with ZOL for 1, 3, 5 and 7 days, after which cells were counted using trypan blue exclusion assay. Following ZOL treatment, U87 and MDA-MD-432 cell numbers decreased.

We determined the effect of NBPs on proliferation of cancer cell lines by alamar blue assay *in vitro* as reported previously [22, 23]; we calculated IC_50_ values for 4 NBPs (Zoledronic acid, Risedronic acid, Alendronate, Ibandronate) in various cancer cell lines. Specifically, we used the breast cancer cell line MDA-MD-43, the GBM cell line U87 and GBM patient-derived primary cell line SK429 (Figure 1B). In all 3 types of cells, our data showed zoledronic acid (ZOL) to be the most potent drug with the lowest IC_50_ value (Figure 1A). We also measured tumor cell numbers on day 0, 1, 3, 5 and 7, with or without ZOL treatment. Without ZOL treatment (control group), U87 and MDA-MD-432 cells numbers increased dramatically; In the treatment group, after incubation with ZOL, we detected attenuation in cell numbers. This inhibition of cell proliferation by ZOL is dose- and time-dependent (Figure 1C).

Further, we tested an additional 4 GBM cell lines, 4 breast cancer cell lines, and 8 GBM patient-derived primary cell lines cultured in NSA stem cell media (sphere culture); ZOL showed a potent inhibitory effect on the growth of these different human cell lines, with IC_50_ values ranging from 3 μM to 37 μM. Importantly, we observed that the inhibitory effect of ZOL was time dependent; after 3- and 5-day treatments, the IC_50_ values for ZOL were larger than 300 and 100 μM respectively and much higher than those following 7-day treatments.

### Zoledronic acid induces tumor cell death but without apoptosis signals

**Figure 2:**
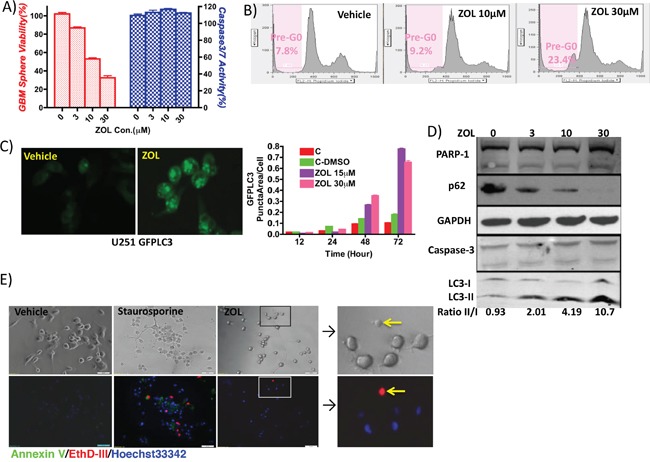
Induction of cell cycle arrest and cell autophagy by ZOL treatment **A.** Cell viability was examined by alamar blue assay and caspase-3/7 activity using Apo-One Kit, following treatment with increasing doses of ZOL (0, 3, 10, and 30 μM). Cell viability decreased but without a detectable apoptotic signal. **B.** Effect of ZOL on cell cycle was studied using flow cytometry and quantifying percentage of pre-G0 cells. ZOL treatment induced cell cycle arrest – pre-phase cells increased from 7.8% (control) to 9.2% (10 μM ZOL) and 23.4% (30 μM ZOL). **C.** ZOL induced autophagosome accumulation showed by U251-GFPLC3 puncta formation in tumor cell, when compared to control cells where GFPLC3 is evenly distributed in the cytoplasm. Cells are visualized by fluorescence microscopy, and the puncta in cells are quantified in 5 randomly selected fields. **D.** Western blotting analysis to detect PARP-1 and caspase 3 (apoptotic signals) and LC3-II/I conversion and p62 degradation (autophagy signals) following ZOL treatment (0, 10, and 30 μM ZOL). **E.** Annexin V staining phosphatidylserine (PS) on the surface of apoptotic cells with green fluorescence. EthD-III stains necrotic cells and late apoptotic cells with red fluorescence. Hoechst33342 stain the entire cell population with blue fluorescence. Staurosporine-treated cells serve as positive control for apoptosis. After ZOL treatment, U87 cells did not demonstrate any apoptotic and necrotic signals.

ZOL attenuated cancer cell viability, as evidenced by a decrease in fluorescence intensity of alamar blue reagent; this effect on cell viability was concentration-dependent (Figure 2A). FACS analysis also showed that the cell population of pre-G_0_ cells increased dramatically, from 7.8% to 23.4% after ZOL treatment (Figure 2B). Unexpectedly however, in cells treated with ZOL at doses as high as 30 μM, we were unable to detect an apoptotic signal. Our fluorescence assay did not detect caspase-3/7 activity; similarly, western blotting could not detect either caspase-3 cleavage or PARP-1 band; furthermore, annexin V staining also showed very weak or negative signal (Figure 2E). In contrast, under the same conditions, we observed strong autophagy signals – conjugation of LC3-I to LC3-II and p62 degradation – by fluorescence microscope and western blotting (Figure 2C and 2D). These findings strongly suggest that autophagy, rather than apoptosis may be the predominant pathway activated following ZOL treatment.

### *In vivo* growth-inhibitory effect of zoledronic acid on malignant glioma xenografts

**Figure 3:**
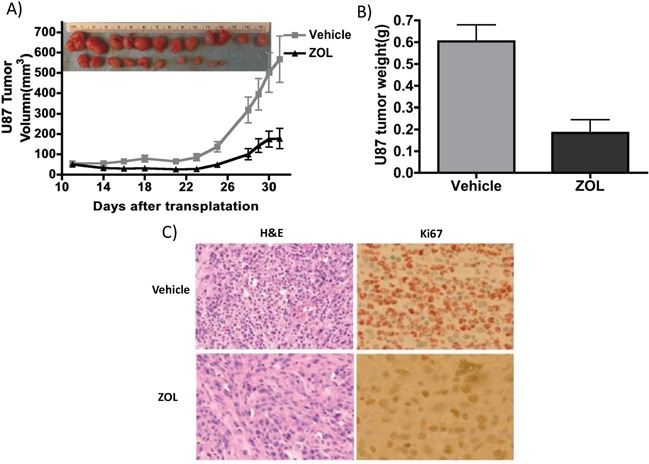
ZOL retards U87 xenografts growth *in vivo* **A.** U87 tumors were implanted subcutaneously in flanks of NSG mice; mice were treated with 0.2 mg/kg ZOL 5 times /week about 3 weeks. Tumor size decreased after ZOL treatment; at day 31 the average size is 567.8±114.3mm3 vs. 178.3±50.4mm3 in vehicle-treated mice (n=12, P<0.01). **B.** Tumor weight was significantly lower following ZOL treatment; the average weight of ZOL-treated tumors was 0.60±0.26g vs. 0.18±0.21g in controls (n=12, P<0.001). **C.** Ki67 staining showed less proliferation cell in tumor after ZOL treatments.

We investigated the effect of ZOL on glioma tumor growth *in vivo*, using subcutaneously implanted U87 xenograft models in immune-compromised mice. We injected ZOL locally around the U87 tumor site, once the tumor was 100-200 mm^3^. Following ZOL treatment, we observed an attenuation in tumor volume, when compared to control animals treated with vehicle alone (Figure 3A and 3B). Injection of 0.2 mg/kg ZOL was effective in inhibiting growth of subcutaneous U87 tumors. This inhibition of proliferating tumor cells *in vivo* was further confirmed by Ki67 staining of tumor cells, after mice were sacrificed following drug treatment (Figure 3C).

### Geranylgeraniol reversed the zoledronic acid effect on tumor cells

**Figure 4:**
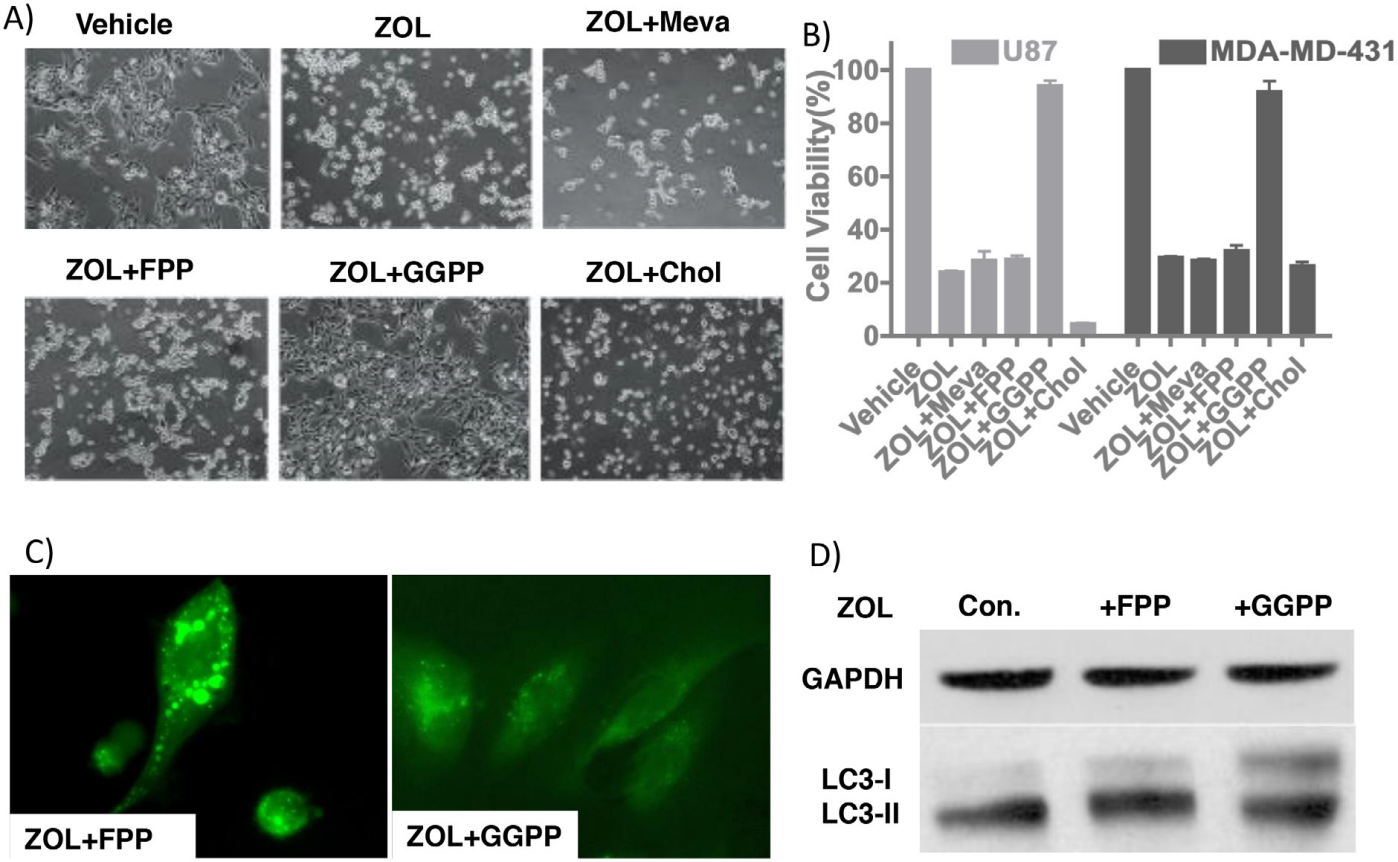
GGPP rescues ZOL-treated tumor cells **A.** Light microscopic images demonstrating morphological changes in U87 cells treated with ZOL alone or in combination with intermediates of the cholesterol pathway. **B.** Cell viability of U87 and MDA-MD-431 cells detected using alamar blue assay following with ZOL alone or in combination with intermediates of the cholesterol pathway shows rescue of ZOL treated cells with GGPP but not with Mevalonate or cholesterol. Mean cell viability of control, ZOL and ZOL+GGPP groups is 103.0%±0.56% vs. 23.9%±2.0% vs. 93.9%±1.9% for U87 cell, 102.4%±1.9% vs. 29.4%±0.56% vs. 91.7%±4.1% for MDA-MD-431 cell. **C.** U251-GFPLC3 puncta formation induced by ZOL rescued after adding GGPP but not by FPP **D.** LC3I/II conversion induced by ZOL rescued by GGPP (Lane +GGPP) but not by FPP (Lane +FPP). U87 treated with ZOL as Control.

As ZOL targets multiple genes in the cholesterol synthesis pathways [15–18], we sought to identify whether specific intermediates from this pathway could rescue the anti-tumor effect of ZOL *in vitro*. After ZOL treatment, geranylgeranyl pyrophosphate (GGPP), but not mevalonate, farnesyl-pyrophosphate (FPP) or cholesterol rescued cell viability and reversed the inhibitors effects of ZOL (Figure 4A, 4B). These data suggest that depletion of GGPP after ZOL treatment plays a critical role in the drug's cytotoxic effect. ZOL treatment also induced autophagy in GBM cells as showed previously; this autophagy was rescued by addition of GGPP but not FPP. In our study, exogenous GGPP but not FPP neutralized the GFPLC3 puncta formation and LC3I/II conversion (Figure 4C, 4D). However, the mechanism by which GGPP protects cancer cell from ZOL treatment is unclear and needs to be further explored. Although these data clearly indicated that GGPP plays an essential role in maintaining cell survive and cell viability, details have not been well characterized.

### RabGGTase but not GGTase-1 response for the zoledronic acid cytotoxic effect

**Figure 5:**
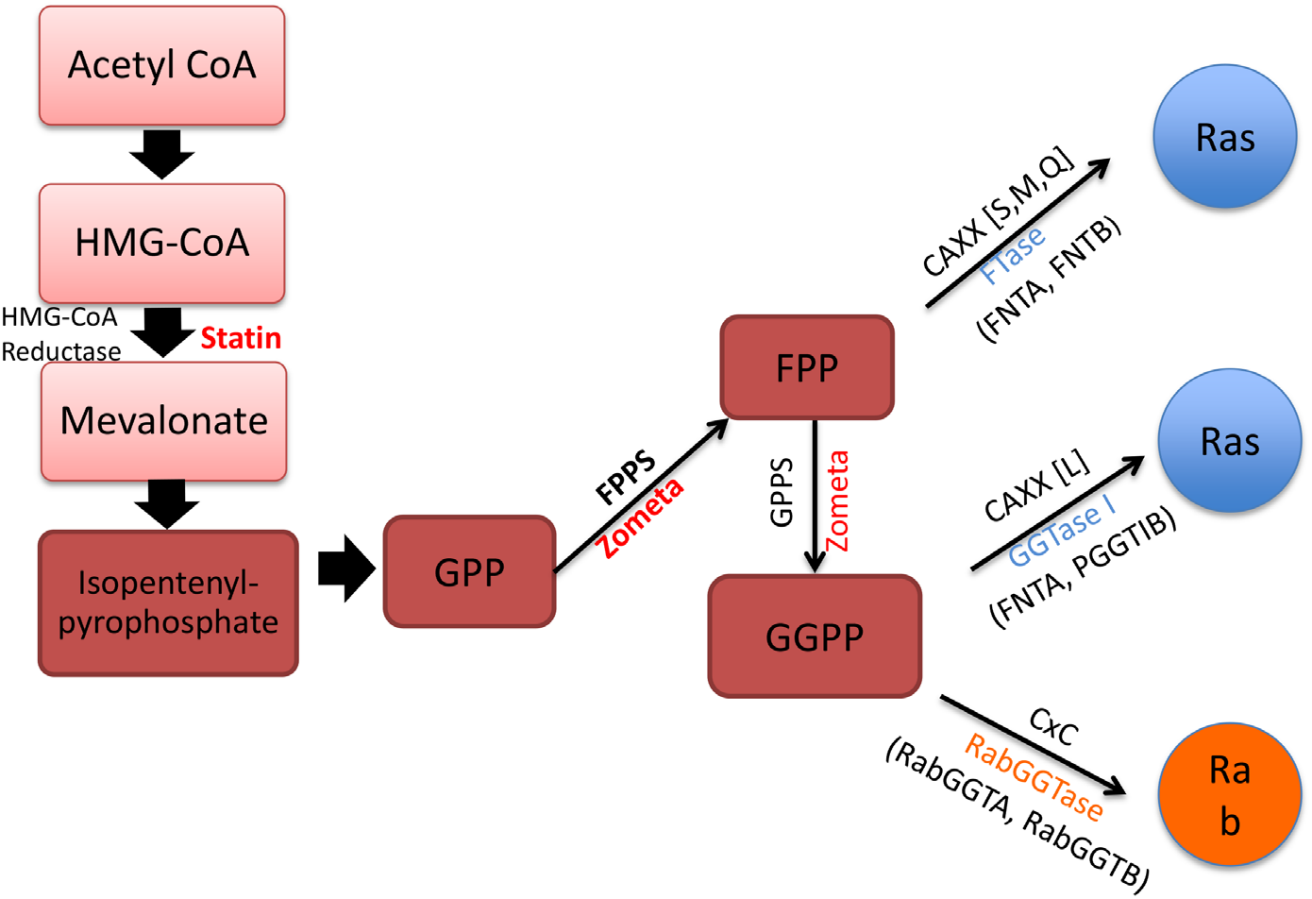
Mevalonate synthesis pathway and related prenyl-transferases This cartoon depicts the sites of action of ZOL and statins targeting the mevalonate synthesis pathway. The intermediates in this pathway, FPP and GGPP also modify Ras and Rab proteins. The prenyltransferases include FTase, GGTase-1 and RabGGTase: FTase encoded by FNTA and FNTB gene, GGTase-1 encoded by FNTA and PGGT1B, and RabGGTase encoded by RabGGTA and RabGGTB.

**Figure 6:**
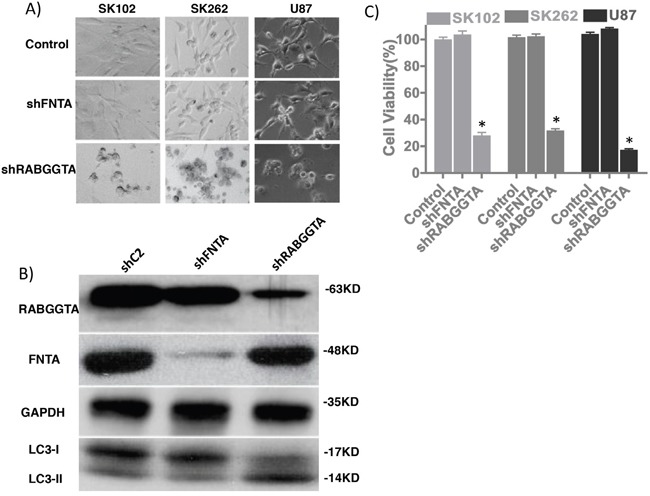
RabGGTA mediates anti-tumor effects of ZOL **A.** Morphological changes after lentiviral transduction of U87 glioma cells and SK262 and SK102 patient-derived GBM cell lines, targeting FNTA and RabGGTA genes. **B.** Western blotting depicting expression of FNTA and RabGGTA following shRNA expression. Decreased RabGGTA expression is associated with LC3-II/LC3-I conversion, very similar to cell after ZOL treatment. **C.** Cell viability in cells following shRNA-induced knockdown of FNTA and RabGGTA demonstrates decreased viability only after RabGGTA knockdown (U87 cell viability 99.3%±4.0% *Vs.* 27.3%±5.1%, p=0.002) but not FNTA (99.3% ±4.0% *Vs.* 103.1%±5.6%, p=0.24). GBM patients' primary cell lines SK102 and SK262 also have similar results.

GGPP is a 20-carbon isoprenoid, an important metabolite produced by the mevalonate pathway. GGPP plays a role in prenylation of several low molecular weight G proteins, including Ras and Rab. Ras proteins are involved in transmitting signals within cells (cellular signal transduction), and Rab proteins are a family of related proteins involved in exocytic and endocytic membrane trafficking. We investigated the role of GGPP in modifying Rab and Ras protein prenylation and their possible contributions in the cytotoxic effect of ZOL. GGPP modifies Ras and Rab by different enzymes (GGTase I and RabGGTase respectively). We blocked the Ras and Rab functions by knockdown of FNTA and RabGGTA subunits (Figure 5). The GBM cell line U87 and 2 primary patient lines (SK102 and SK262) were transduced with lentiviral vectors to silence the FNTA and RabGGTA genes by shRNA. To maximum the efficacy of lentiviral infection, the primary patient cells were cultured adherent to laminin-1 coating plates. We found that down-regulated RabGGTA proteins (Figure 6B) induced decreased cell viability, but FNTA knockdown had no such effect (Figure 6). These data indicate that Rab prenylation likely plays an essential role in cancer cell viability, likely related to GGPP function.

## DISCUSSION

To meet the urgent need for providing more effective glioblastoma therapies, we screened and investigated FDA-approved drugs used for other non-cancer indications, on GBM cells, including patient-derived primary GBM cells. In previous studies, we have demonstrated that statin drugs targeting the cholesterol synthesis pathway (also named as mevalonate pathway) can effectively inhibit growth of human cancer cells *in vitro* and *in vivo* [22, 23]. In the current study, we tested another class of FDA-approved drug, NBPs that target the cholesterol synthesis pathway as well. Here, we confirm using established and patient-derived cancer cells that these drugs, specifically ZOL effectively inhibits the proliferation of human cancer cells *in vitro* and tumor growth *in vivo*. Interestingly, ZOL also induced tumor cell cycle arrest as reported [24, 25, 26] and cellular autophagy. In addition, GGPP can rescue cell viability. GGPP was also able to rescue the effect of statins, as demonstrated in our previous study [22, 23]. Our data presented here indicate that the cholesterol synthesis pathway is important to maintain cancer cell viability, by providing cells with intermediates in the pathway, as well as cholesterol, the final product.

In general, dysregulation of the cholesterol synthesis pathway promotes cell transformation and it has been demonstrated that HMG-CoA reductase and additional genes function as candidate metabolic oncogenes [27]. This has provided the rationale for the use of cholesterol synthesis pathway inhibitors as effective anti-cancer therapeutics, in addition to their use in preventive cardiovascular diseases. For the latter, inhibition of the cholesterol synthesis pathway reduces levels of its final product, cholesterol, which in turn is responsible for its primary clinical benefit. But adding cholesterol back after statin or NBP treated tumor cells does not rescue tumor cell viability; this suggests that other intermediate(s) (not cholesterol) may be more important in maintaining tumor cell viability. Further, depletion of intracellular intermediates of the cholesterol synthesis pathway in cancer cells is time-dependent and the maximum inhibition effect is observed in 3-5 days for statins and 5-7 days for NBPs. Hence, the key for repurposing cholesterol synthesis inhibitors as effective cancer therapeutic agents is the availability of long-lasting drugs that can effectively deplete cholesterol pathway intermediates reservoir in cancer cells.

Amongst the multitude of intermediates, farnesylpyrophosphate (C15) and geranylgeranylpyrophosphate (C20) are extremely important biological molecules as they are required for post-translational modification (prenylation) of approximately 2% mammalian proteins, including oncogene Ras and Ras-homologous (Rho) GTPases. These proteins, which play pivotal roles in signal transduction, require C-terminal prenylation for correct intracellular localization and function [28, 29]. Abnormality of protein prenylation is documented in several human diseases, including cancer, progeria and parasite infections. Protein prenylation is catalyzed by three known protein prenyltransferases in eukaryotic cells: protein farnesyltransferase (FTase), protein geranylgeranyl-transferase type I (GGTase-I), and geranylgeranyltransferase type II (GGTase-II or Rab GGTase) (Figure 5). FTase adds C15 (farnesyl) isopreniod moiety to target proteins, whereas GGTase-1 and Rab GGTase use C20 (gernylgeranyl) group. FTase and GGTase-1 share a common alpha subunit [30]. Protein prenyl-transferases are generally highly selective for isoprenoid diphosphate substrate (FPP or GGPP), but a single mutation in FTase can act as a molecular switch in the process of isoprenoid selectivity [31]. However, no studies have examined specific switches in the Rab GGTase.

Unlike the eukaryotes cells, the nematode *Caenorhabditis elegans* possesses a very unique cholesterol synthesis pathway that is deficient in its sterol synthesis branch but has the other noncholesterol intermediate products. In *C. elegans*, following RNAi screening, it was shown that silencing RABGGTA and RABGGTB induces larval lethality; however, targeting FNTA resulted in egg-laying abnormal but with no effect on survival; In addition, that targeting *C. elegans* FNTB that has no phenotypic changes [32]. These results are consistent with our data that inhibit RABGGTase but not FTase and GGTase-1 is critical for cancer cell death. GGPP also plays an essential role in maintaining normal brain function and neuronal survival, including learning, as demonstrated in mouse models [33, 34]. *In vitro*, GGPP induces cell apoptosis and cell cycle arrest in tumor cells [35, 36]. But the exact role of GGPP has not been elucidated. In our previous study, we found that GGPP can rescue cancer cells after statin treatment [23]. Similarly, here we show that GGPP broadly rescues cancer cells following ZOL treatment. *In vivo*, a genomic mutation in RABGGTA gene decreases its enzyme activity by ~70% and impairs enzyme's function such as prenylation of Rab proteins in *gummetal* mouse model. Although the defective *gummetal* mouse has prolonged bleeding, thrombocytopenia, and reduced platelet counts, the mouse is otherwise normal [37, 38]. As Rab proteins are essential for normal cellular function, it is believed that complete deficiency of RabGGTase may be lethal; however, in mice, inhibition of RabGGTase activity still showed restricted phenotype. Due to a lack of appropriate analytical methods, the activity of prenylation enzymes in normal cells and cancer cells have not been studied, as gene transcription and protein expression level maybe not a good predictor. The study of *gummetal* mice indicate that normal cells still have a great change to well-tolerant when using reagents targeting RabGGTase in cancer therapy. As RabGGTase is a good target for cancer therapy, the effects of specific inhibitors of RabGGTA are under investigation; these inhibitors may represent a novel approach for cancer therapy [39, 40, 41].

The Rab small GTPases are a member of the Ras superfamily. Approximately 70 types of Rab proteins have now been identified in humans. Rab are localized in the intracellular membranes and they are crucial regulators of membrane traffic pathways in both differentiated and neoplastic cells [42]. There are abundant reports that alternative Rab expression plays a role in carcinogenesis. The protein Rab27a plays a crucial role in transporting lysosomes from the microtubule-organizing center (MTOC) to the plasma membrane, and enhanced Rab27a expression is closely associated with pancreatic cancer; furthermore, Rab27a-expressing hepatocellular carcinoma patients have poor survival, and invasive/metastatic potential of human breast cancer cells directly correlates with Rab27a expression [43, 44, 45, 46].Rab6 is located within the Golgi apparatus and it regulates protein trafficking; Rab6-mediated lung cancer growth is promoted by miR-5100 [47, 48]. Similarly, Rab7 plays key roles in lysosome biogenesis and has been demonstrated to be directly associated with melanoma progression [49, 50]. In spite of these studies on Rab proteins, the specific Rab(s) involved in the ZOL effect need to be identified. Our current study on ZOL provides evidence that inhibition of Rab prenylation may be an appropriate target for cancer therapy. The cancer therapeutic effects of NBPs may due to their ability to suppress Rab prenylation but of which targets and to what extent is still an open question that needs further study.

In conclusion, we demonstrate the effective anti-tumor activity of ZOL in breast cancer and glioma cell lines, *in vitro* and in xenograft mouse models. Importantly, this effect of ZOL is mediated at least in part through depletion of GGPP, an intermediate of the cholesterol synthesis pathway, which in turn acts by affecting Rab protein(s) prenylation. These data demonstrate the mechanism of anti-cancer effects of an FDA-approved drug class, bisphosphonates that can be repurposed. Importantly, data from this study also point to Rab prenylation as a possible novel target for cancer drug discovery and development.

## MATERIALS AND METHODS

### Reagents

Four nitrogen-containing BPs (NBP) obtained from commercial sources: Zoledronic acid monohydrate (SML0223, Sigma), risedronic acid sodium salt (R521500, Toronto Research Chemicals), alendronate monosodium trihydrate, ibandronate monosodium monohydrate (A4515, I0502, LKT Laboratories). We prepared 10 mM stock solution in PBS solution. Intermediate products of cholesterol synthesis were purchased from Sigma: mevalonolactone (M4667), farnesyl pyrophosphate ammonium salt (F6892), geranylgeranyl pyrophosphate ammonium salt (G6025), squalene (S3626), cholesterol solution (S5442), geranylpyrophosphate (G6772), and isopentenyl pyrophosphate triammonium salt solution (I0503).

### Cell culture

Established breast cancer (MCF-7, MDA-MB-231, MDA-MB-432, MDA-MB-435) and glioblastoma cell lines (A172, LN229, U87, U118, U251, U373) were grown in DMEM medium supplemented with 10% FBS at 37°C in a humidified 5% CO_2_ atmosphere. Patient-derived stem cell-like GBM cell lines (GBM4, GBM8, SK102, SK262, SK429, SK748, SK987, SK1035) were cultured in complete NeuroCult Proliferation Medium with hEGF and hFGF (#05702, #02653 and #02654, Stemcell Technologies). Institutional Review Boards of the UC San Diego Human Research Protections Program reviewed and approved this project (IRB # 100936), in accordance with the requirements of the Code of Federal Regulations on the Protection of Human Subjects. The IRB granted a waiver of informed consent for the recruitment component of this project. Adherent SK102, SK262 was cultured in Laminin-1 coated dish as previous described [22, 23].

### Cell proliferation assay

All cells were seeded into 96-well plates at a density of 2,000 cells/well. GBM cell were seeding at 2000/well in 96 well plates overnight. The N-BP drugs were serially diluted using the culture media and cells were incubated for the times mentioned. Alamar Blue (#BUF012B, AbDSerotec) was then added, according to the manufacture's manual, directly to the culture medium and the fluorescence signal was read at 560/590 after 4 hours to determine the number of viable cells (Infinite M200, Tecan Group Ltd.). The IC_50_ values were calculated using a commercial software (Prism^®^, Graphpad Software, La Jolla, CA).

### Cell growth inhibition assay

Tumor cells were added to 6-well plates overnight at a density of 5×10^4^/well and then treated with 1, 3 and 10 μM zoledronic acid or vehicle (PBS). At day 1, 3, 5, or 7 after treatment, the cell numbers in each well were counted by trypan blue assays according to standard protocols. All experiments were repeated in triplicates.

### Western-blot

LC3, p62 (autophagy), PARP-1, caspase-3 (apoptosis) and GAPDH (as loading control), FNTA and RabGGTA were detected by western blotting following drug treatment. GBM cell were treated with 3, 10, or 30 μM zoledronic acid for 72 h, the cell lysates were then harvested, and 10-30 μg whole protein was loaded onto or 6%-12% or 14% SDS-PAGE gel (for LC3). Proteins transferred to PVDF membrane and probed with primary antibodies (L8918 Sigma, #8025 Cell signaling, sc-25780 Santa Cruz, #9662 Cell signaling, GTX627408 GeneTex, GTX46222 GeneTex, AV49162 Sigma). The resultant protein bands were visualized by a supersignal kit (#1856136, Thermo Scientific) after incubation with HRP-labeled secondary antibodies. The results were recorded on autoradiography film (#165-1496, Kodak) and the films were scanned after development. The ratios of band intensities of LC3-II/LC3-I were calculated by NIH Image-J software.

### Flow cytometric analysis of cell cycle

U87 cells were plated at a density of 5×10^5^ cells in 6-cm dishes overnight. Cells were washed twice with PBS, followed by fresh media with 10, 30 μM zoledronic acid. After 48 hours, the cells were fixed in 70% ethanol in PBS and stored at −20°C for 24 hours. Propidium iodide staining of DNA was carried out and analyzed via flow cytometry (Canto, BD FACS) to determine the distribution of cells in different phases of the cell cycle with the aid of ModFit LT software (version 3.0).

### Inoculation of tumor and drug treatment

The *in vivo* study was performed on xenograft mouse models, using nude mice to evaluate the drug effect to inhibit tumor growth. 2×10^6^ U87 cells were subcutaneously transplanted at the right and left flanks. Initial tumor growth was monitored every 3 days. Drug administration was initiated when the tumors reached a size of 100-120 mm^3^. Mice were randomly regrouped into 2 groups of 6 mice each, without significant difference in tumor volume before drug treatment. The mice were treated with either PBS as control or zoledronic acid. Zoledronic acid was administered as a subcutaneous injection at 0.2 mg/kg dose, about 1 cm from the tumor, once per day, on a 5-days-on, 2-days-off schedule. Tumor size and mice weights were measured 2 times per week. All mice were sacrificed after tumors reach over 1 cm in diameter in the control group. Tumor volumes were calculated as (length×width×width/2). After sacrificing the mice, all tumors were dissected and weighed. The animal protocol was approved by UCSD Institutional Animal Care and Use Committee (IACUC).

### Quantifying GFPLC3 puncta by fluorescence microscopy

Stable U251 cell lines expressing GFPLC3 (GFP fusion with LC3) or GFP (retroviral transduction) was selected as described previously [22, 23]. U251/GFPLC3 cells or U251/GFP cells were seeded to 5×10^4^/well in 24-well plates, and zoledronic acid was added to final concentrations of 15 and 30 μM for U251. The cells were visualized at 12, 24, 36, 48 and 60 hrs. Fluorescent images were taken at 5 random locations in each well. The GFPLC3 clusters were quantified using the particle counting function in Metamorph software (Molecular Devices LLC, Sunnyvale, CA, United States). The number of cells and the GFPLC3 cluster in the cells were quantified using Cell Profiler software (Molecular Devices LLC, Sunnyvale, CA, United States). The number of LC3 clusters of an image was normalized using the number of cells in that image. The mean and standard error of mean of the 5 images (>100 cells) were calculated.

### Gene knock-down

Confluent 293T cells and transfected with pCMV-VSVG, pCMV-dR8.9 packaging plasmids with SHC002 (pKLO.1 non-target control plasmid, sigma SHC002), pKLO.1-ΔFNTA, pKLO.1-ΔRABGGTA (TRCN0000034587 and TRCN0000036190, Sigma). After 48 and 72 h, the lentivirus suspension was collected and used to infect U87, SK102 and SK262 cells. Cells were observed using a light microscope daily. The collected cells with infected control or knockdown shRNA at 72 h had no significant morphological changes. The FNTA and RABGGTA protein expression was confirmed to decrease at 48-72 h after infection by western-blot.

### Statistical analysis

The unpaired two-tailed t-test was used to calculate statistical significance. P<0.05 was set as the level of significance.
